# Central Acting Hsp10 Regulates Mitochondrial Function, Fatty Acid Metabolism, and Insulin Sensitivity in the Hypothalamus

**DOI:** 10.3390/antiox10050711

**Published:** 2021-04-30

**Authors:** Kristina Wardelmann, Michaela Rath, José Pedro Castro, Sabine Blümel, Mareike Schell, Robert Hauffe, Fabian Schumacher, Tanina Flore, Katrin Ritter, Andreas Wernitz, Toru Hosoi, Koichiro Ozawa, Burkhard Kleuser, Jürgen Weiß, Annette Schürmann, André Kleinridders

**Affiliations:** 1 Junior Research Group Central Regulation of Metabolism, German Institute of Human Nutrition Potsdam-Rehbruecke, Arthur-Scheunert-Allee 114-116, 14558 Nuthetal, Germany; kristina.wardelmann@dife.de (K.W.); rath@uni-potsdam.de (M.R.); sabine_bluemel@yahoo.de (S.B.); mareike.schell@dife.de (M.S.); rhauffe@uni-potsdam.de (R.H.); tanina.flore@dife.de (T.F.); katrin.ritter@dife.de (K.R.); 2 German Center for Diabetes Research (DZD), Ingolstaedter Land Str. 1, 85764 Neuherberg, Germany; juergen.weiss@ddz.de (J.W.); schuermann@dife.de (A.S.); 3 Department of Experimental Diabetology, German Institute of Human Nutrition Potsdam-Rehbruecke, Arthur-Scheunert-Allee 114-116, 14558 Nuthetal, Germany; 4 Department of Molecular and Experimental Nutritional Medicine, Institute of Nutritional Science, University of Potsdam, 14558 Nuthetal, Germany; 5 Department of Molecular Toxicology, German Institute of Human Nutrition Potsdam-Rehbruecke, Arthur-Scheunert-Allee 114-116, 14558 Nuthetal, Germany; jose.castro@i3s.up.pt; 6 Aging and Aneuploidy Laboratory, IBMC, Instituto de Biologia Molecular e Celular, Universidade do Porto, 4200-135 Porto, Portugal; 7 Instituto de Investigação e Inovação em Saúde, Universidade do Porto, 4200-135 Porto, Portugal; 8 Institute of Pharmacy, Freie Universität Berlin, Königin-Luise-Str. 2+4, 14195 Berlin, Germany; fabian.schumacher@fu-berlin.de (F.S.); burkhard.kleuser@fu-berlin.de (B.K.); 9 Department of Toxicology, University of Potsdam, Arthur-Scheunert-Allee 114-116, 14558 Nuthetal, Germany; 10 Department of Molecular Epidemiology, German Institute of Human Nutrition Potsdam-Rehbruecke, Arthur-Scheunert-Allee 114-116, 14558 Nuthetal, Germany; andreas.wernitz@dife.de; 11 Department of Clinical Pharmacology, Faculty of Pharmaceutical Sciences, Sanyo-Onoda City University, 1-1-1 Daigaku-dori, Sanyo Onoda, Yamaguchi 756-0884, Japan; hosoi@rs.socu.ac.jp; 12 Department of Pharmacotherapy, Graduate School of Biomedical and Health Sciences, Hiroshima University, 1-2-3 Kasumi, Minami-ku, Hiroshima 734-8551, Japan; ozawak@hiroshima-u.ac.jp; 13 German Diabetes Center (DDZ), Leibniz Center for Diabetes Research, Institute for Clinical Biochemistry and Pathobiochemistry, 40225 Duesseldorf, Germany

**Keywords:** brain insulin signaling, mitochondria, oxidative stress, fatty acid metabolism

## Abstract

Mitochondria are critical for hypothalamic function and regulators of metabolism. Hypothalamic mitochondrial dysfunction with decreased mitochondrial chaperone expression is present in type 2 diabetes (T2D). Recently, we demonstrated that a dysregulated mitochondrial stress response (MSR) with reduced chaperone expression in the hypothalamus is an early event in obesity development due to insufficient insulin signaling. Although insulin activates this response and improves metabolism, the metabolic impact of one of its members, the mitochondrial chaperone heat shock protein 10 (Hsp10), is unknown. Thus, we hypothesized that a reduction of Hsp10 in hypothalamic neurons will impair mitochondrial function and impact brain insulin action. Therefore, we investigated the role of chaperone Hsp10 by introducing a lentiviral-mediated Hsp10 knockdown (KD) in the hypothalamic cell line CLU-183 and in the arcuate nucleus (ARC) of C57BL/6N male mice. We analyzed mitochondrial function and insulin signaling utilizing qPCR, Western blot, XF96 Analyzer, immunohistochemistry, and microscopy techniques. We show that Hsp10 expression is reduced in T2D mice brains and regulated by leptin in vitro. Hsp10 KD in hypothalamic cells induced mitochondrial dysfunction with altered fatty acid metabolism and increased mitochondria-specific oxidative stress resulting in neuronal insulin resistance. Consequently, the reduction of Hsp10 in the ARC of C57BL/6N mice caused hypothalamic insulin resistance with acute liver insulin resistance.

## 1. Introduction

Altered energy homeostasis, insulin resistance, and mitochondrial abnormalities are features of type 2 diabetes (T2D). Insulin resistance in the brain causes metabolic disorders since lack of insulin receptor signaling in the brain causes obesity and hyperleptinemia, and alters hepatic glucose production [1,2]. Identifying novel signaling pathways affecting brain insulin signaling can help to reveal new potential treatment options for metabolic disorders. Mitochondrial dysfunction strongly associates with insulin resistance. For example, deteriorations of mitochondria in diabetes include altered biogenesis, dynamics, increased reactive oxygen species (ROS) generation, or an altered mitochondrial proteome [3]. The mitochondrial chaperone Hsp60 facilitates its protein folding function mainly in cooperation with its co-chaperone Hsp10 in a 1:1 stoichiometry. To ensure this balance, the expression of both chaperones in the mitochondria is controlled by a bidirectional promoter [4]. Together, they form a ubiquitously expressed protein complex assembled into two heptameric rings. These structures entail an inner cavity enabling an ATP-dependent folding of mitochondrial proteins. The co-chaperone Hsp10 forms a lid structure, modulates Hsp60 activity, and controls the ATPase cycle during the event of refolding proteins [5,6]. This chaperone complex folds more than 250 proteins, including the antioxidative enzyme superoxide dismutase 2 (SOD2), and has been shown to interact with more than 300 proteins [7,8,9]. Further, this chaperone complex belongs to the mitochondrial stress response (MSR), a mitochondrial-nuclear signaling pathway, which helps mitochondria to cope with stress and reinstate proper mitochondrial function [10]. Thus, Hsp60/Hsp10 is crucial to protect mitochondria and thereby cells against oxidative stress, by, e.g., supporting a proper antioxidative response. We could demonstrate that inhibiting the protein folding capacity of mitochondrial matrix proteins, by decreasing the expression of mitochondrial chaperone Hsp60, impairs insulin action in the hypothalamus due to excessive amounts of superoxide radical formation [11]. Mutations of Hsp60 even cause spastic paraplegia 13 and a fatal demyelinating leukodystrophy [12,13], showing the immense impact of mitochondrial protein folding on brain function.

Despite its prominent role as co-chaperone, Hsp10 exhibits also Hsp60 independent effects with extramitochondrial and nonfolding functions [14], e.g., with roles in immunomodulation [15] and presumably in neurite outgrowth and nucleic acid metabolism in the brain [16], indicative of a role for brain development and homeostasis. Recently, a mitochondrial reduction of Hsp10 has been shown to be an early event in synucleinopathies while overexpression of Hsp10 counteracts disease phenotypes and improves mitochondrial function [17]. Further, HSPE1 (Hsp10 gene) mutation is linked to neurodegenerative diseases [18], supporting the hypothesis that a dysregulation of the MSR is detrimental to brain health. We could identify insulin action as a modulator of the MSR in the hypothalamus [19]. Therefore, mitochondrial dysfunction, at least induced by dysregulation of Hsp60 along with oxidative stress, represents a cause and consequence of hypothalamic insulin resistance [20]. Yet, mitochondrial dysfunction impacts cellular energy metabolism and proper clearance of ROS, as well as fatty acid metabolism [21]. Although the brain does not depend on fatty acid metabolism as its energy source, fatty acid sensing in the hypothalamus seems to control metabolism [22,23]. Increases in saturated long-chain fatty acids, diverse sphingolipids, or ceramides are linked to insulin resistance, indicating that an increase in overall fatty acid and lipid metabolism is detrimental for insulin action. In contrast, mild impairment of hypothalamic fatty acid metabolism can also improve glucose homeostasis [24], suggesting a complex interplay of hypothalamic fatty acid metabolism and peripheral metabolism, which presumably depends on the exact fatty acid and lipid profile. Interestingly, patients deficient for Hsp60 expression show clear signs of altered fatty acid homeostasis, indicating that a dysregulated Hsp60/Hsp10 complex, as observed in diet-induced insulin resistance [25], might also impact hypothalamic fatty acid metabolism. Thus, hypothalamic mitochondrial dysfunction might have a broad control of metabolism by affecting oxidative stress, fatty acid metabolism, and insulin action, thereby dysregulating central and peripheral metabolism [20,26]. We hypothesized that a reduction of Hsp10 in hypothalamic neurons will impair mitochondrial function and cellular metabolism, and impact brain insulin action. Therefore, we investigated the regulation of Hsp10 in T2D, in particular, its impact on mitochondrial function and insulin action in the hypothalamus. We show that Hsp10 is decreased in brain samples of T2D mice and is inducible by leptin in vitro. Its reduction is sufficient to cause mitochondrial oxidative stress and dysfunction, altered fatty acid metabolism, and insulin resistance in hypothalamic neurons. Furthermore, reducing Hsp10 expression in the hypothalamus of C57BL/6N mice causes hypothalamic insulin resistance with acute liver insulin resistance, identifying a novel role for Hsp10 in propagating insulin sensitivity in vivo.

## 2. Materials and Methods

All chemicals were of analytical or higher grade and obtained from local providers, unless otherwise stated.

### 2.1. Cell Culture

CLU-183 cells (mHypoA-2/23 CLU-183) and SH-ObRb (human neuroblastoma SH-SY5Y cell line stably transfected with Ob-Rb long isoform of leptin receptor) cells [27] were cultivated with DMEM GlutaMAX (Gibco, Carlsbad, CA, USA) supplemented with 1 mM sodium pyruvate (Gibco), 10% fetal bovine serum (FBS) (Pan Biotech, South Africa) and 1% penicillin–streptomycin (Gibco). All cell cultures were maintained at 37 °C with 5% CO_2_.

#### 2.1.1. Infection with Lentiviral Particles

CLU-183 cells were infected using lentiviral transduction particles with pLKO.1 plasmid either containing shRNA targeting Hspe1 gene (HSPE1 MISSION shRNA Lentiviral Transduction Particles, SHCLNV-NM_008303; Sigma-Aldrich, St. Louis, MO, USA) or control transduction particles targeting no known mammalian genes (MISSION pLKO.1-puro Non-Mammalian shRNA Control Transduction Particles, SHC902V; Sigma) according to the manufacturer’s instructions. To enhance the infection efficiency of the virus, 12 µg/mL hexadimethrine bromide (polybrene) was added for incubation overnight. After 16 h incubation, fresh cultivating media was added. After an additional 8 h, 5 µg/mL of appropriate selection antibiotic (puromycin) was added to the media to kill untransfected cells. The selection was carried out for at least one week. The following experiments were mostly conducted with three mixed clones of Hsp10 KD and control.

#### 2.1.2. Stimulation Experiments

CLU-183 cells were serum starved (DMEM GlutaMAX, 1 mM sodium pyruvate, 1% penicillin–streptomycin, without FBS) for 3 h prior to 5 min stimulation with 1 nM insulin (Sigma). SH-ObRb cells were serum starved (DMEM GlutaMAX, 1 mM sodium pyruvate, 1% penicillin–streptomycin, 0.1% BSA) for 3 h prior to stimulation with 10 nM human leptin (Peprotech, Hamburg, Germany) for 6 h. The different stimulations were stopped with ice-cold PBS.

### 2.2. Animal Studies

For the experiments, 10-week-old male C57BL/6N wildtype mice were obtained from Charles River Laboratories and the animals remained in groups of 4–6 in the animal facility of the German Institute of Human Nutrition for one week (habituation) before the stereotactical injection. In addition, 11-week-old male BKS.Cg-Dock7m+/+LeprdbJ (db/db) mice were obtained from Charles River Laboratories and remained in the animal facility before dissection of the organs. All animal study and care protocols were in accordance with the animal welfare committees of the German Institute of Human Nutrition and approved by the local authorities (State Agency of Environment, Health, and Consumer Protection, LUGV, Brandenburg, Germany). Animals were kept on a 12 h dark–light cycle with free access to food and water (ad libitum) in a temperature-controlled room (22 ± 1 °C).

### 2.3. Stereotactical Injection of Lentiviral Particles

Briefly, 11-week-old male C57BL/6N were anesthetized by i.p. injection of ketamine (50 mg/kg body weight (BW))/Medetomidine hydrochloride (0.8 mg/kg BW). In addition, the mice were injected with Buprenorphine (0.1 mg/kg BW) to prevent postoperational pain. The injection site for the operation was shaved, and to prevent drying of the eyes during the operation, eye cream (Bepanthen, Bayer Vital GmbH, Leverkusen, Germany) was applied. The mouse was fixed into the stereotactical apparatus, and the site of the operation disinfected with Octenisept before drilling two small holes into the cranium with a dental driller. The cannulas were placed for the bilateral injection of lentiviral particles. The coordinates for the injection into the arcuate nucleus of the hypothalamus were the following and calculated using the mouse brain atlas from “Paxinos” [28]: Bregma −1.45 mm; middle line ±0.25 mm; below the surface of the skull −5.85 mm. The injection of 0.375 µL (1 × 10^6^) of lentiviral particles in 10 min per side was performed with a pump. The lentiviral particles used contain pLKO.1 plasmid producing either an shRNA targeting Hspe1 gene or control transduction particles targeting no known mammalian genes (same constructs as used in cell culture experiments). The operation site was stitched with a 6–0 Glycolon (Resorba Medical GmbH, Nürnberg, Germany). The mice were injected with an antagonist for the anesthesia, Atipamezol hydrochloride (1.245 mg/kg BW) to wake them up and put into their separate housing cage containing easily accessible water bowls along with a bowl of Carprofen-Medigel^®^/Clear H2O sucralose gel supplemented with carprofen (5 mg/kg/day) for 2 days before surgery and 3 days after surgery under the surveillance of the experimenter.

### 2.4. Insulin Injection into Vena Cava

To assess local insulin sensitivity, mice were starved for 16 h and anesthetized by injecting i.p. Ketamine (100 mg/kg BW)/Medetomidine (0.8 mg/kg BW). Saline or 5 U of insulin was injected directly into the vena cava. Then, organs were harvested after 2 min (liver) and 10 min (brain).

### 2.5. Final Anesthesia

Animals were euthanatized by inhalation of isoflurane, followed by heart puncture, and/or cervical dislocation. Organs were harvested and brain areas were dissected using a mouse brain matrix (Zivic Instruments). For the improvement of immunohistochemistry staining of the brain, the animals were perfused with 0.9% NaCl, followed by the perfusion with 4% paraformaldehyde (PFA) for fixation. Before perfusion, the mice were euthanatized by injecting i.p. Ketamine (100 mg/kg BW)/Xylazine (16 mg/kg BW). Afterward, the thorax was opened to inject a rounded needle into the left ventricle of the heart. The right ventricle of the heart was opened and 0.9% NaCl solution was steadily injected (10 mL/min) for about 2 min to wash out the vessels. Afterward, 4% PFA solution was steadily injected for fixation of the tissue for about 5 min.

### 2.6. Cryostat-Sectioning and Immunofluorescence

For this part, 4% PFA-perfused brains were treated with a sucrose gradient from 10 up to 30% sucrose in PBS before being completely covered in Tissue-Tek^®^ OCT™ Compound (Sakura Finetek Germany GmbH, Umkirch, Germany) and frozen at −80 °C. The Tissue-Tek covered brain was attached to the sectioning table of the Cryostat with additional Tissue Tek, and 10 µm sections were performed. After sectioning, the slices were attached to superfrost microscope slides and dried overnight at room temperature (RT). The area of interest of the brain slices was marked with a PAP pen.

Cells were directly grown on multiwell chamber slides (Sarstedt, Nürmbrecht, Germany). For fixation, the cells were covered with 4% PFA for 15 min at RT.

The specimen was blocked for 1 h (cells) or 3–4 h (brain slices) at RT (blocking buffer: 1× PBS, 2% FCS, 0.3% Triton-X). After blocking, the diluted primary antibody (primary antibody: Hsp10 from Santa Cruz sc-2095, 1:40 in antibody dilution buffer: 1× PBS, 1% BSA, 0.3% Triton-X) was added to the area of interest. The slides were sealed with parafilm to prevent drying and incubated for 24 h (cells) or 72 h (brain slices) at 4 °C. The slides were incubated with diluted fluorescent secondary antibody (1:500 in antibody dilution buffer: 1× PBS, 1% BSA, 0.3% Triton-X) for 1h at RT in the dark. Finally, 20–30 µL DAPI stain with Fluoromount G was added. The pictures were taken with the confocal microscope LSM 780 (ZEISS, Oberkochen, Germany) and the analysis was performed using ZEN software (version 2.3) from ZEISS.

### 2.7. DNA/RNA Isolation

DNA Isolation from 4 × 10^4^–7 × 10^7^ cells was performed with Invisorb^®^ Spin Tissue Mini Kit (Stratec) according to the protocol supplied by the manufacturer. Total RNA was extracted from 5–20 mg tissue or 4 × 10^5^–7 × 10^5^ cells using a ReliaPrep RNA Tissue Miniprep System (Promega) or RNeasy Mini Kit (Qiagen, Hilden, Germany) following the manufacturer’s manual, including DNase I treatment.

### 2.8. Analysis of Gene Expression by Quantitative PCR

Overall, 100–1000 ng of RNA from tissue and cells were reverse transcribed in 20 µL using Oligo(dT)15 primers (Promega), Random primers (Promega), Thermo Scientific™ dNTP-Set, and M-MLV Reverse Transkriptase (Promega). Real-time PCR was performed using the GoTaq qPCR master mix (Promega), gene-specific primers (200 nM each, obtained from Sigma) (Supplementary Materials Table S1), and 10 ng cDNA. Fluorescence was monitored and analyzed in the ViiA 7 Real-Time PCR System (Applied Biosystems, Foster City, CA, USA). Gene expression was calculated according to the ΔΔCT method using Tbp (TATA-box binding protein) or Chdh1 (choline dehydrogenase) as the reference gene. The specificity of SYBRGreen primers was confirmed by melting curve analysis.

### 2.9. Fractionation

#### 2.9.1. Mitochondrial Isolation from Tissue (Brain)

The desired brain area (e.g., hypothalamus) was freshly isolated into 5–10 mL buffer for cell mitochondria isolation (IBC) buffer (200 mM Sucrose, 1 mM EGTA/Tris pH 7.4, 10 mM Tris/MOPS pH 7.4, cOmplete™ Protease Inhibitor). The sample was homogenized via “pottering” 10× at 1000 rpm on ice. The homogenate was centrifuged at 1000× *g* for 10 min at 4 °C (pellet = unbroken cells), and the supernatant was afterward centrifuged at 17,000× g for 15 min. The supernatant contained the cytoplasm-enriched fraction, and the pellet contained the mitochondria-enriched fraction, which could be resuspended in resuspension buffer (250 mM Sucrose, 10 mM MOPS-KOH, 80 mM KCl, 5 mM MgCl_2_, cOmplete™ Protease Inhibitor) in the appropriate volume, depending on the brain area (200–500 µL).

#### 2.9.2. Mitochondrial Isolation from Cells

At least 1 × 10^7^ cells (15cm dish) were detached by adding 1x trypsin/EDTA and incubated for 3 min at 37 °C. The cell suspension was centrifuged for 3 min at 338 rcf at RT. The cell pellet was resuspended in 1 mL fractionation buffer (250 mM Sucrose, 10 mM Tris pH 7.5, 1 mM EDTA). The cell suspension was homogenized via “pottering” 6× at 1000 rpm on ice. The homogenate was centrifuged at 1000× *g* for 10 min at 4 °C (pellet = unbroken cells), and the supernatant was afterward centrifuged at 17,000 *g* for 15 min. The supernatant contained the cytoplasm-enriched fraction, and the pellet contained the mitochondria-enriched fraction, which can be resuspended in resuspension buffer (250 mM Sucrose, 10 mM MOPS-KOH, 80 mM KCl, 5 mM MgCl_2_, cOmplete™ Protease Inhibitor) in the appropriate volume depending on the cell number (20–50 µL).

### 2.10. Western Blot Analysis

Western blotting was performed on 10–40 µg total protein lysates loaded on 8–15% SDS–PAGE gels and transferred to PVDF membranes (GE HealthCare Life Science, Amersham, UK) for 150 min at 90 V. Following the transfer, the membrane was incubated with ponceau staining solution (0.2% Ponceau S, 3% trichloroacetic acid, 3% sulfosalicylic acid) for detection of total protein content, and membranes were blocked for 1 h in starting block buffer (Thermo Scientific #37543). After blocking, membranes were probed with primary antibody overnight at 4 °C. Antibodies used were pSAPK/pJNK (Thr183/Tyr185) (Cell Signaling, Cambridge, UK #9251, 1:1000), SAPK/JNK (Cell Signaling #9258, 1:1000), Hsp10 (Santa Cruz Biotechnology, Dallas, TX, USA sc-20958, 1:200), Hsp60 (Cell Signaling #4870, 1:1000), VDAC (Cell Signaling #4866 1:1000), pAMPKα (T172) (Cell Signaling #2535, 1:1000), AMPKα (Cell Signaling #5832, 1:1000), SOD2 (Cell Signaling #13141, 1:2000); pDRP1 (S637) (Cell Signaling #4867, 1:1000), DRP1 (Cell Signaling #8570, 1:1000), pIRS1 (S1101) (Cell Signaling #2385, 1:1000), pIRS1 (S636/639) (Cell Signaling #2388; 1:1000), pIRS1 (Y608/612) (Merck Millipore, Burlington, MA, USA #09-432, 1:1000), IRS1 (BD, Heidelberg, Germany 611394, 1:1000), pAKT (T308) (Cell Signaling #13038, 1:1000), AKT (Cell Signaling #9272, 1:1000), PEPCK (Cell Signaling #12940, 1:1000), pIRE1α (S724) (Novusbio, Abington, UK NB100-2323, 1:1000), IRE1α (Cell Signaling #3294, 1:1000), Total OXPHOS Rodent WB Antibody Cocktail (Abcam #ab110413, 1:2000), GFAP (Abcam, Cambridge, UK #7260), 3-Nitrotyrosine (Abcam #ab110282, 1:1000), β Actin+HRP (Sigma-Aldrich A3854, 1:10,000). Membranes were incubated with peroxidase-conjugated secondary antibodies (anti-rabbit Dianova #711-065-152, 1:10,000, anti-mouse Dianova #715-065-150, 1:10,000; or anti-rabbit Cell Signaling #7074S, anti-mouse Cell Signaling #7076S, 1:2000) at room temperature for 1 h. Specific bands were detected by chemiluminescence assay (WesternBright ECL Biozym 541005X) using the ChemiDoc Touch Imaging System (BioRad, Munich, Germany). For removing the phospho-epitope of a protein and run the total antibody, membranes were incubated for 20–30 min in stripping buffer (Restore^TM^ PLUS Western Blot Stripping Buffer Thermo Scientific #46430) at RT, reblocked for 30 min, and reprobed. In the end, membranes were probed with β-Actin, serving as an internal loading control for Western blotting when applicable or stained with FastGreen as the loading control. Band intensities were quantified via densitometric analysis using Image Lab 5.2.1 software (Biorad).

### 2.11. Protein Carbonylation Assay

Protein carbonyl groups were detected with an antibody after protein derivatization, as previously described [29]. Briefly, after ponceau staining, the membrane was washed with water and then equilibrated in 1× TBS + 20% methanol for 5 min. Following that, the membrane was washed for 5 min with 10% HCl and incubated for derivatization in 5 mM 2,4-dinitrophenylhydrazine (DNPH) in 10% HCl for 10 min. Afterward, excess DNPH was removed by washing twice for 5 min with 10% HCl. The final step consisted of washing the membrane 5× 5 min with 50% methanol and 1× 5 min with 1× TBS. After briefly washing with MQ water, the membrane was blocked in starting block for 1 h, then washed three times with 1× TBS-T, and probed with primary antibody anti-DNP (Sigma, D9656, 1:10,000) at 4 °C. Intensities of total signals per the whole lane were quantified in comparison to the whole lane of the protein loading control (ponceau staining) and analyzed via densitometric analysis using Image Lab 5.2.1 software.

### 2.12. Analyses of Mitochondrial Morphology

Cells were seeded onto glass-bottom plates and stained with 100 nM MitoTracker Deep Red FM (Invitrogen) for 30 min in HBSS media without phenol red (Gibco). After washing, the cells were put into a CO_2_ chamber while taking videos and pictures using the confocal microscope LSM 780 (ZEISS).

### 2.13. Seahorse Assay

For the determination of mitochondrial respiration, the Seahorse XF96 Flux Analyzer (Agilent, Santa Clara, CA, USA) was used. Cells were seeded at a density of 5000 cells per well one day prior to the experiment on 96 multiwell plates, specifically designed for the Seahorse flux analyzer. Four wells were left without cells for blank measurement. The plate was incubated overnight at 37 °C with 5% CO_2_. The sensor cartridge is hydrated with Seahorse XF Calibrant Buffer (Agilent) at 37 °C without CO_2_ at least 16 h before the experimental run. The flux analyzer measures the oxygen consumption rate (OCR) as well as the extracellular acidification rate (ECAR). On the day of the experimental run, cells were washed once carefully with PBS, assay medium (minimum DMEM with 10 mM glucose, 1 mM pyruvate, 2 mM glutamine) was added and the measurement of the mitochondrial respiration started in the flux analyzer. Prior to that, the compounds inhibiting different subunits of the ETC were prepared freshly in assay medium and pipetted into designed ports for the serial injection. Final compound concentrations used: 2 mM Oligomycin, 1 mM FCCP, 1 mM Rotenone/2 mM Antimycin A. The injections and measurements were performed according to the manufacturer’s protocol (MitoStress Test Kit, Agilent), and for normalization, total protein content was determined after the run. Data were evaluated using Wave software (Agilent).

### 2.14. Electron Microscopy

Confluent neurons were fixed by immersion in 2.5% glutaraldehyde in 0.19 M sodium cacodylate buffer at pH 7.4, postfixed in 1% reduced osmium tetroxide [30] in Aqua Bidest, and afterward stained with 2% uranyl acetate in maleate buffer, pH 4.7. The specimens were dehydrated in graded ethanol and embedded in epoxy resin (Epon 812), as described by Luft [31] and modified by Reale [30]. Ultrathin sections were picked up onto Formvarcarbon-coated grids, stained with lead citrate, and viewed in a transmission electron microscope (TEM 910; Zeiss Elektronenmikroskopie, Oberkochen, Germany).

### 2.15. Fatty Acid (FA) Analysis of Cell Total Lipids by Gas Chromatography (GC)

Analysis of FA spectra of cell total lipids was performed with a strongly modified method using extraction with tert-butyl methyl ether/methanol, hydrolysis and methylation with boron trifluoride/methanol, and subsequent analysis by GC [32,33,34]. 8–10 × 10^6^ cells were harvested in 1 mL buffered phosphate. Modifications of the analysis method were previously published in [35]. FA composition of cell total lipids was expressed as area percentage of each FA relative to total area of all detected FA: C14:0; C15:0; C16:0; C16:1n7c; C17:0; C18:0; C18:1n9c; C18:1n7c; C18:2n6c; C20:0; C18:3n3; C20:1n9; C20:3n9; C20:3n6; C20:4n6; C20:5n3; C22:0, C23:0, C24:0; C24:1n9; C22:4n6; C22:5n6; C22:5n3; C22:6n3.

### 2.16. Sphingolipid Quantification by HPLC–MS/MS

Cells were incubated with 250 μM palmitic acid (16,16,16-d3) from Cortecnet (Voisins-le-Bretonneux, France) applied as a BSA complex for 16 h at 37 °C. Afterward, cells were pelleted, washed, and lysed in an aqueous buffered solution. Aliquots were subjected to lipid extraction using 1.5 mL methanol/chloroform (2:1, *v:v*), as described [36]. The extraction solvent contained d7-dihydrosphingosine (d7-dhSph), d7-sphingosine (d7-Sph), d7-sphingosine 1-phosphate (d7-S1P), C17-ceramide (C17:0 Cer) and C16-d31-sphingomyelin (C16:0 d31-SM) (all Avanti Polar Lipids, Alabaster, USA) as internal standards. Chromatographic separations were achieved on a 1260 Infinity HPLC (Agilent Technologies, Waldbronn, Germany) equipped with a Poroshell 120 EC-C8 column (3.0 × 150 mm, 2.7 µm; Agilent Technologies). MS/MS analyses were carried out using a 6490 triple-quadrupole mass spectrometer (Agilent Technologies) operating in the positive electrospray ionization mode (ESI+) [37]. MS/MS parameters for detection of canonical and deuterated sphingolipids are given in Supplementary Table S2. Quantification was performed with MassHunter Software (Agilent Technologies). Determined lipid amounts were normalized to the actual protein content (determined via Bradford assay) of the cell lysate aliquot used for extraction.

### 2.17. Statistical Analysis

Statistical analysis was carried out using GraphPad Prism 9 (GraphPad Software, La Jolla, CA, USA). All Data are represented as mean ± SEM. Unpaired two-tailed Student’s *t*-test was used for comparing two normal distributed groups. A two-way ANOVA was performed for interactions between treatment or genotype between groups and Sidak’s multiple comparisons when appropriate. Graphs and statistical analysis were generated with GraphPad Prism 7 Software.

## 3. Results

### 3.1. Metabolic Dysregulation of Hsp10 in T2D

**Figure 1:**
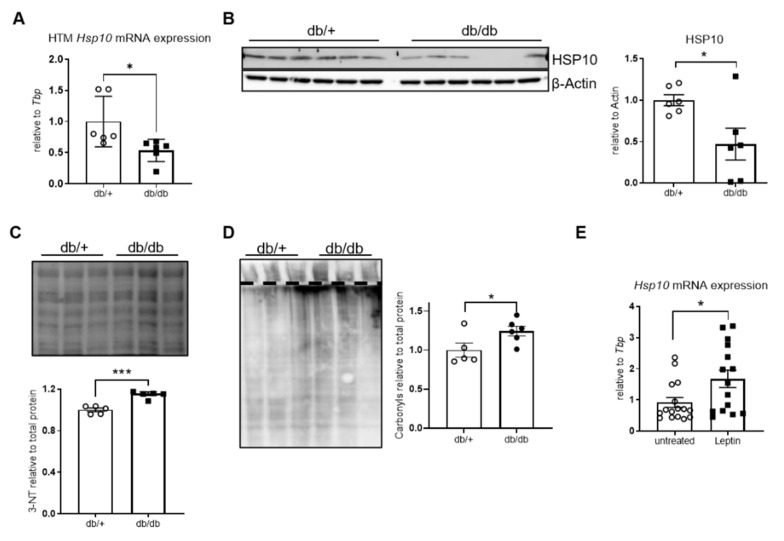
Metabolic regulation of Hsp10 and its dysregulation in a model of type 2 diabetes (T2D). (**A**) mRNA expression analysis of Hsp10 in the HTM of db/db mice and (**B**) Western blot and densitometric analysis of mitochondrial fraction from db/db mice brains. *n* = 6 for db/+ and *n* = 6 for db/db. (**C**) Representative Western blot (3 vs. 3) and densitometric analysis of protein oxidation (3-nitrotyrosine) of all analyzed db/db mice brains. *n* = 5 for db/+ and *n* = 6 for db/db. (**D**) Representative Western blot (3 vs. 3) and densitometric analysis of protein oxidation (carbonylation) of db/db mice brains. *n* = 5 for db/+ and *n* = 6 for db/db. (**E**) mRNA expression of Hsp10 of serum-starved control and leptin-treated SH-ObRb cells for 6 h, *n* = 18. *, *p* < 0.05, ***, *p* < 0.001 after two-tailed Student’s *t*-test. All data are presented as mean ± SEM.

To investigate whether Hsp10 was dysregulated in the brains of diabetic mice, we determined the expression profile of Hsp10 in db/db mice brains. Hsp10 gene and protein expression was reduced by ~50% in obese, hyperglycemic db/db mice, compared to db/+ controls (Figure 1A,B). This was associated with increased oxidative stress in the brain as evidenced by elevated 3-nitrotyrosine and protein carbonylation in brain samples of diabetic mice (Figure 1C,D; metabolic data in Figure S1A,B). Since db/db mice are leptin resistant, we next analyzed whether leptin was able to induce Hsp10 gene transcription.

To test leptin’s effect on Hsp10 expression in vitro, we used the transgenic cell line SH-SY5Y (neuroblastoma cells) overexpressing the long isoform of leptin receptor (ObRb). Treating these cells with leptin caused a 70% increase in Hsp10 gene expression (Figure 1E), indicating that Hsp10 is a leptin-inducible chaperone, as already described for Hsp60 [11].

### 3.2. Hsp10 KD in Hypothalamic Cells Induces Mitochondrial Dysfunction and Changes Mitochondrial Dynamics

**Figure 2:**
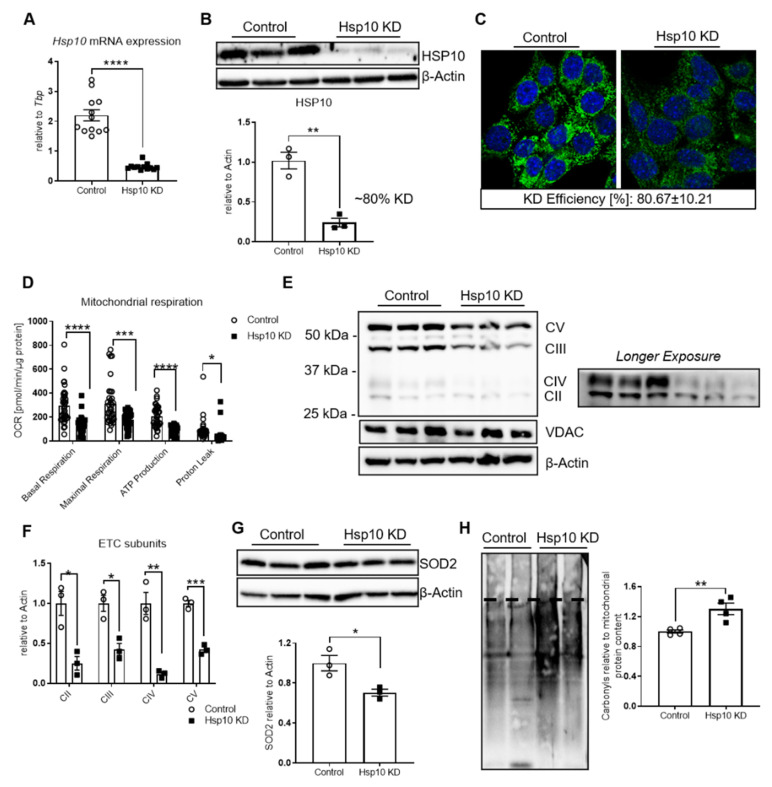
Lentiviral-induced KD of Hsp10 in hypothalamic cells induces mitochondrial dysfunction with mitochondria-specific oxidative stress. (**A**) mRNA expression of Hsp10 of control and Hsp10 KD CLU-183 cells, *n* = 12. (**B**) Protein expression of HSP10 and densitometric analysis, *n* = 3. β-Actin was used as loading control. Representative blot from three different experiments. (**C**) Representative pictures of immunofluorescence staining of control and Hsp10 KD cells. Green = Hsp10, blue = DAPI, *n* = 5–6 pictures per condition. (**D**) Analysis of bioenergetics profile of control and Hsp10 KD cells using the Seahorse Bioflux Analyzer XF96. *n* = 10–12 per condition. Normalization to total protein. (**E**) Protein expression of mitochondrial OXPHOS complex subunits of CII-CV of control and Hsp10 KD cells. VDAC served as a control for mitochondrial content, β-Actin served as a loading control n=3. (left) Representative blot from three independent experiments. (right) Longer exposure of the same representative blot for better illustration of expression of CII and CIV. (**F**) Densitometric analysis of Western blot shown in (**E**). (**G**) Protein expression of SOD2 and densitometric analysis, *n* = 3. β-Actin was used as loading control. Representative blot from three independent experiments. (**H**) Representative Western blot (2 vs. 2) and densitometric analysis (4 vs. 4) of protein carbonylation of the mitochondrial fraction of control and Hsp10 KD cells. Representative blot from two different experiments, *n* = 4. *, *p* < 0.05, **, *p* < 0.01, ***, *p* < 0.001, **** *p* < 0.0001 after two-tailed Student’s *t*-test. All data are presented as mean ± SEM.

To address the impact of Hsp10 in hypothalamic neurons on cellular metabolism, we employed a lentiviral-mediated knockdown strategy using an shRNA directed against Hsp10 and a nontargeted control in the murine hypothalamic cell line CLU-183. This approach resulted in a ~80% knockdown of Hsp10 on mRNA and protein levels, which was further verified by immunofluorescence staining (Figure 2A–C). This reduction did not cause a compensatory upregulation of different members of the MSR (Figure S2A), as had been shown for mitochondrial proteases of the MSR [38]. To assess mitochondrial function, we used the Seahorse Bioflux Analyzer. Knockdown of Hsp10 (Hsp10 KD) caused a strong reduction of mitochondrial activity with a 55% and 43% reduction in basal and maximal respiration, with additional reduction in ATP production by 56% and an altered proton leak by 42% (Figure 2D, original profile of this experiment in Supplementary Figure S2B). Mitochondrial dysfunction with reduced ATP production and expression of key enzymes of β-oxidation can activate the energy sensor of the cell, 5’ AMP-activated protein kinase (AMPK). Assessing the activated form of AMPK by Western blotting for Y172 phosphorylation of the α-subunit of AMPK, we identified 40% increased phosphorylation of AMPK in Hsp10 KD cells, compared to control (Figure S2C). Since mitochondrial dysfunction can cause neuroinflammation and alter autophagy, we assessed suppressor of cytokine signaling 3 (SOCS3) gene expression as a marker of increased inflammation and p62 and LC3 levels as markers for autophagy. There was no difference in SOCS3 mRNA expression (Figure S2D), nor in p62 protein expression or an increase in LC3 II formation between control and Hsp10KD neurons (Figure S2E), suggesting that this Hsp10 knockdown does not cause neuroinflammation or alter autophagy in our experimental setting. In summary, these data indicate that the reduction of Hsp10 severely impacts mitochondrial activity and energy metabolism of hypothalamic neurons.

Next, we assessed whether dysfunctional mitochondria due to decreased Hsp10 levels modified mitochondrial protein expression and caused further mitochondrial abnormalities. Mitochondrial content, as measured by the ratio of mitochondrial to genomic DNA content, was unaltered with unchanged mitochondrial transcription factor A (Tfam) levels but enhanced peroxisome proliferator-activated receptor gamma coactivator 1-α (Pgc1α) expression (Figure S2F,G). No change in mitochondrial DNA (mitoDNA) does not exclude effects on mitochondrial count or mass since there can be 1–10 copies of mitoDNA per mitochondrion [39]. Hsp10 reduction caused decreased protein levels of subunits of the electron transport chain complexes II, III, IV, and V, indicating that altered mitochondrial proteostasis is responsible for mitochondrial dysfunction (Figure 2E,F). The reduction in protein level could not solely be explained by decreased mRNA levels pointing to the crucial role of Hsp10 in folding mitochondrial matrix proteins (Figure S2H). Next, we examined the effect of reduced Hsp10 levels on oxidative stress formation. The reduction of Hsp10 resulted in a ~30% reduction of SOD2 protein but not mRNA levels, indicating decreased folding of SOD2 in the absence of Hsp10 (Figure 2G, Figure S2I). In line with this observation, Hsp10 KD neuronal cells exhibited increased oxidative stress in mitochondria since protein carbonylation was only increased in mitochondrial fractions (Figure 2H, Figure S2J).

**Figure 3:**
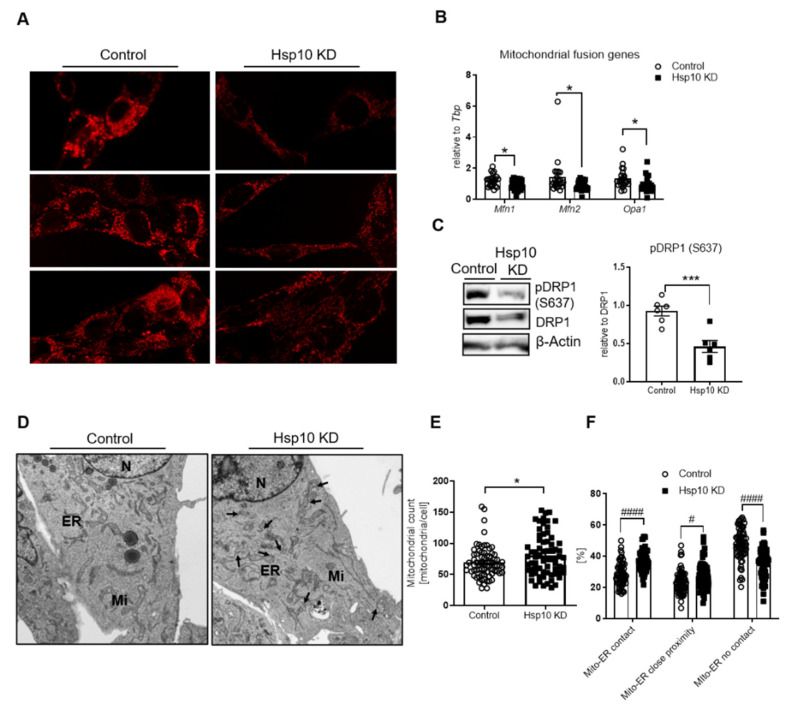
Lentiviral-induced KD of Hsp10 in hypothalamic cells alters the mitochondrial network. (**A**) Confocal immunofluorescent analysis of control and Hsp10 KD cells using MitoTracker Deep Red. Note: Mitochondria of Hsp10 KD are more fragmented and the signal is reduced. (**B**) mRNA expression of mitochondrial fusion genes Mfn1, Mfn2, and Opa1 of control and Hsp10 KD CLU-183 cells. *n* = 24. (**C**) Representative Western blot of pDRP1 (S637) (1 vs. 1) and densitometric analysis of all tested control and Hsp10 KD samples (6 vs. 6). (**D**) Representative electron microscopy analysis of control and Hsp10 KD cells (80% KD). Black arrows indicate mito–ER contact sites. (**E**) Analysis of mitochondrial count and (**F**) mitochondria–ER contact sites as well as mitochondria and ER without contact of Hsp10 KD cells in comparison to control cells, *n* = 69–70. *, *p* < 0.05, ***, *p* < 0.001 after two-tailed Student’s *t*-test. #, *p* < 0.05, ####, *p* < 0.0001 after two-way ANOVA and Sidak’s multiple comparisons test. All data are presented as mean ± SEM.

Mitochondrial dysfunction and oxidative stress are often accompanied by alterations of mitochondrial dynamics. We used MitoTracker Deep Red to visualize mitochondrial morphology and activity. This compound integrates into mitochondria and can be used for quantification of the mitochondrial membrane potential [40]. Hsp10 KD cells exhibited reduced MitoTracker Deep Red intensity compared to control, confirming decreased mitochondrial function. Further, the mitochondrial network morphology of Hsp10 KD cells revealed smaller and more mitochondria displayed by increased punctuation, indicating an increased mitochondrial fission phenotype (Figure 3A). Mitochondrial dynamics are regulated by mitochondrial fusion proteins mitofusin 1 (Mfn1), Mfn2, and mitochondrial dynamin-like GTPase (Opa1) as well as mitochondrial fission protein dynamin-related protein (Drp1). In line with increased mitochondrial fission, Hsp10 KD cells exhibited reduced gene expression of *Mfn1*, *Mfn2*, and *Opa1* in the presence of activated Drp1, as evidenced by decreased inhibitory S637 phosphorylation (Figure 3B,C). In addition, we used electron microscopy to visualize mitochondrial morphology and identified an increased mitochondrial number, supporting our observation of mitochondrial fission (Figure 3D,E). For instance, it has been shown that the endoplasmic reticulum (ER) is marking division sites for mitochondrial fission and is involved in the recruiting process of Drp1 oligomers, demonstrating the importance of mito–ER contact sites for mitochondrial fission [41,42]. Interestingly, mitochondria were in closer proximity to and exhibited increased contact sites with ER in Hsp10 KD cells (Figure 3D, indicated by black arrows, quantification in Figure 3F). Since increased contact sites could be a possible signaling mechanism of stress between the two compartments [43], we tested if this interaction results in a modulated ER stress response. This was, however, not the case in Hsp10 KD cells, shown by the unaltered amount of spliced X-box binding protein 1 (Xbp1), unchanged expression of CCAAT-enhancer-binding protein homologous protein (Chop), and activating transcription factor 4 (Atf4); it neither activated protein kinase/endoribonuclease IRE1α (Figure S3A,B) nor caused this reduction of Hsp10 cell death since mRNA levels of B-cell lymphoma 2 (Bcl2) and Bcl-2-associated protein (Bax) were indistinguishable from controls (Supplementary Figure S3C).

### 3.3. Hsp10 KD in Hypothalamic Cells Exhibit Dysregulated Lipid Metabolism

**Figure 4:**
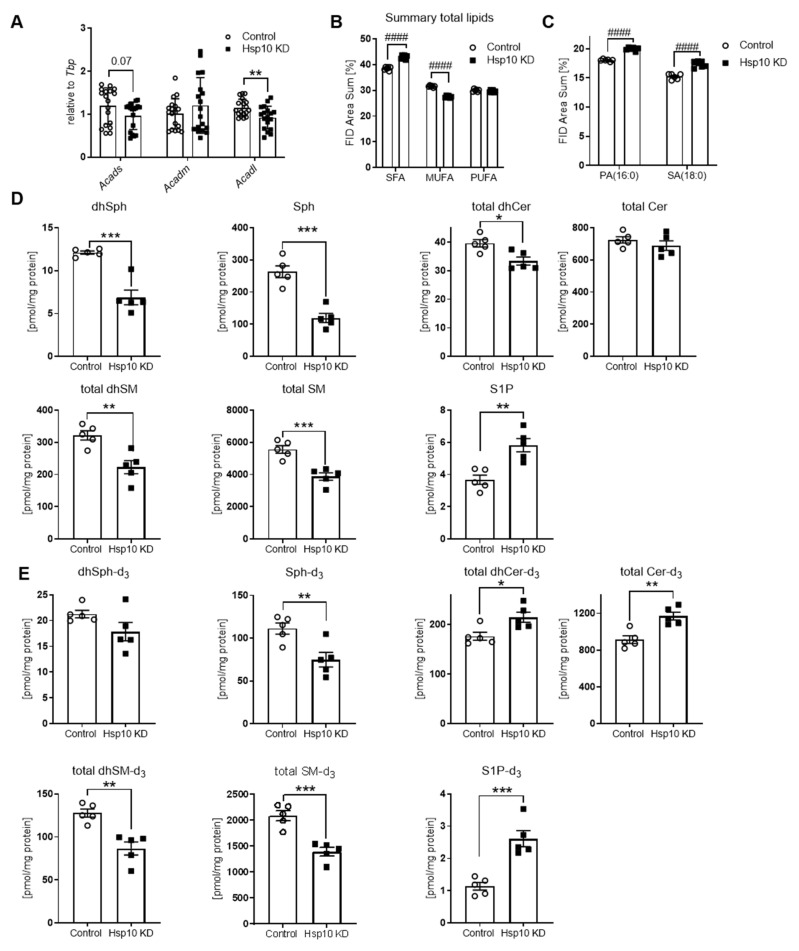
Hsp10 KD in hypothalamic cells induces dysregulated lipid metabolism. (**A**) mRNA expression of acyl–CoA dehydrogenases Acads, Acadm, Acadl of control and Hsp10 KD cells, *n* = 18. (**B**) Fatty acid analysis of cell total lipids in Hsp10 KD and control cells; shown is the area summary of saturated fatty acids (SFA), monounsaturated fatty acids (MUFA), and polyunsaturated fatty acids (PUFA) in percent, *n* = 6. (**C**) Fatty acid analysis of cell total lipids in Hsp10 KD and control cells; shown is the increase in area summary of palmitic acid (PA) and stearic acid (SA) in Hsp10 KD cells in percent, compared to control, *n* = 6. (**D**) Quantification of sphingolipids by HPLC–MS/MS; shown are dihydrosphingosine, sphingosine, total dihydroceramides, ceramides, dihydrosphingomyelin, sphingomyelin, sphingosine-1-phosphate and (**E**) dihyrosphingosine-d_3_, sphingosine-d_3_, total dihydroceramide-d_3_, ceramide-d_3_, diyhdrosphingomyelin-d_3_, sphingomyelin-d_3_ and sphingosine-1-phosphate-d_3_ of Hsp10 KD and control cells *n* = 5. *, *p* < 0.05, **, *p* < 0.01, ***, *p* < 0.001 after two-tailed Student’s *t*-test. ####, *p* < 0.0001 after two-way ANOVA and Sidak’s multiple comparisons test. All data are presented as mean ± SEM.

Fatty acid oxidation is an important aspect of mitochondrial function. Mitochondrial folding targets of the Hsp60/Hsp10 complex which are important for β-oxidation are acyl–CoA dehydrogenases, SCAD, and MCAD [44,45]. Hsp10 KD caused a significant reduction of Acadl and a trend to decreased Acads mRNA levels, while Acadm expression was unaltered, compared to control (Figure 4A). When assessing the lipid profile of Hsp10 KD cells compared to control, Hsp10 KD cells were characterized by a 4% increase in saturated fatty acids (SFA) with decreased monounsaturated fatty acids (MUFA), while polyunsaturated fatty acid (PUFA) levels were not changed, indicating a dysfunctional lipid metabolism (Figure 4B). In particular, there was a 2% increase of pro-inflammatory long-chain SFAs, palmitate (PA), and stearate (SA) in the total lipid fraction of Hsp10 KD cells, in comparison to control (Figure 4C). We further assessed sphingolipids in control and Hsp10KD neurons that have been incubated with deuterated palmitate-d_3_, which, together with serine, represent the initial building blocks of the sphingoid base backbone, in order to differentiate between the intrinsic and de novo formed pool of sphingolipids. This analysis revealed reduced levels of canonical dihydrosphingosine (dhSph), dihydroceramide (dhCer), dihydrosphingomyelin (dhSM), and sphingomyelin (SM) in Hsp10 KD neurons, indicating a possibly affected sphingolipid de novo synthesis in response to the Hsp10 knockdown. Total ceramides (Cer) were unchanged (Figure 4D). Contradictory, de novo formed dhSph-d_3_ was unaltered between control and Hsp10KD cells, and amounts of dhCer-d_3_ and Cer-d_3_ were even more abundant in Hsp10KD neurons. Consistently between intrinsic and de novo formed sphingolipids, levels of dhSM and SM were significantly lower in Hsp10 KD neurons (Figure 4D,E). Strikingly, Hsp10 KD cells showed an almost 55% decrease in sphingosine (Sph) (33% decrease Sph-d_3_) and a 60% increase in S1P (60% increase S1P-d_3_) (Figure 4D (canonical), E (de novo)), as well as an increased *Sphk1* and *Sphk2* mRNA expression along with an upregulation of Sphingosine-1-phosphate receptor 2-5 (*S1pr2-5*) (Figure S3D,E). Elevated levels of palmitate, stearate, and S1P have been shown to be instrumental in inducing insulin resistance, suggesting that an altered neuronal lipid and fatty acid metabolism might impact insulin action and energy metabolism.

### 3.4. Hsp10 KD Induces Cellular Insulin Resistance

**Figure 5:**
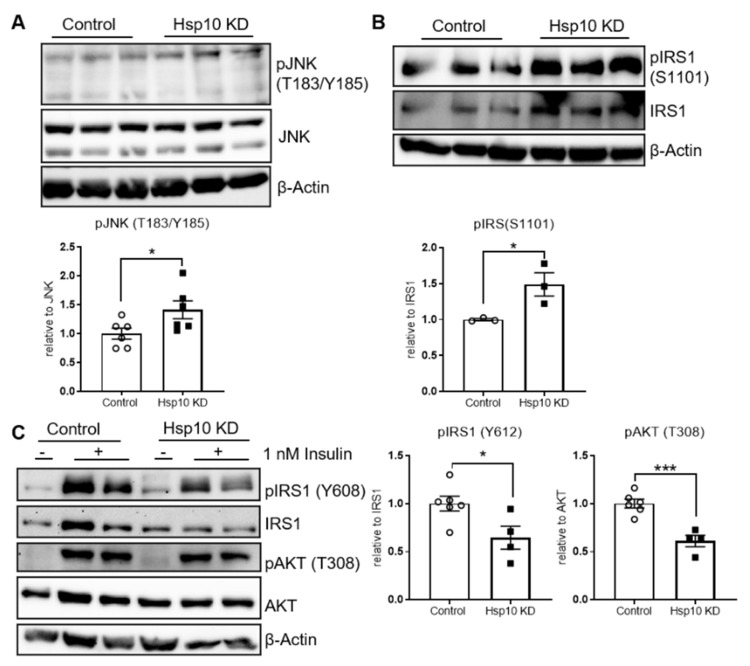
Hypothalamic Hsp10 KD increases pJNK phosphorylation and induces insulin resistance. (**A**) Representative Western blot and pooled densitometric analysis (upper band at 54 kDa) of pJNK in control and Hsp10 KD cells under basal conditions, *n* = 6. (**B**) Western blot analysis of phosphorylation state of IRS1 (S1101) of control and Hsp10 KD cells under basal conditions. Representative blot of three different experiments, *n* = 3. (**C**) Western blot analysis of phosphorylation of IRS1 and AKT of control and Hsp10 KD cells under acute stimulation with 1 nM insulin for 5 min. Representative blot of three different experiments and pooled densitometric analysis (4 vs. 4). *, *p* < 0.05, ***, *p* < 0.001 after two-tailed Student’s *t*-test. All data are presented as mean ± SEM.

Since mitochondrial dysfunction and altered lipid metabolism are linked to inflammation and insulin resistance, we investigated whether a reduction of Hsp10 affected these responses. Hsp10 KD neuronal cells exhibited increased activation of c-Jun N-terminal kinase (JNK) with 40% elevated phosphorylation levels at T183/Y185, compared to control (Figure 5A). Increased serine phosphorylation of insulin receptor substrate 1 (IRS1) proteins reveals a signature of insulin resistance. Since novel antibodies against JNK-dependent S307 phosphorylation of IRS1 did not work properly for Western blotting, we tested S1101 phosphorylation as a marker of insulin resistance [46]. Hsp10 KD cells exhibit insulin resistance under basal conditions, as evidenced by a 48% increase of IRS1 S1101 phosphorylation (Figure 5B), and also showed acute insulin resistance, compared to control. Thus, 1nM insulin stimulation after 3 h serum deprivation demonstrated a 35% reduction of IRS1 Y612 phosphorylation and a concomitant 40% reduction of T308 phosphorylation of protein kinase B (AKT) in Hsp10 KD cells, compared to control (Figure 5C). To understand whether activation of JNK was responsible for the observed insulin resistance, we treated Hsp10 KD cells with JNK inhibitor SP600125. However, this treatment was unable to reverse Hsp10 KD-induced insulin resistance (Figure S3F). Further, treatment with antioxidants such as N-acetylcysteine (NAC) or the mitochondria-specific antioxidant MitoTEMPO did not improve insulin sensitivity in Hsp10 KD cells (data not shown). In summary, the reduction of Hsp10, as observed in db/db mice brains, is sufficient to induce mitochondrial dysfunction, mitochondria-specific oxidative stress, and insulin resistance in hypothalamic neurons.

### 3.5. Hsp10 KD in the ARC of C57BL/6N Mice Induces Hypothalamic Insulin Resistance

**Figure 6:**
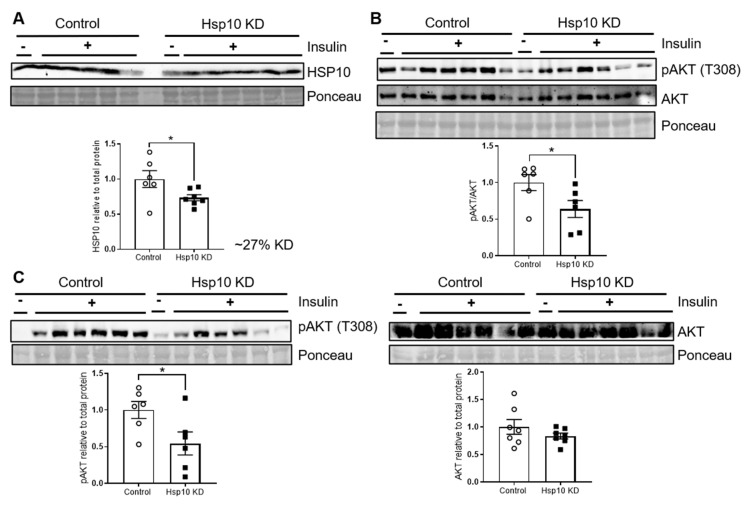
Hsp10 KD in the ARC reduces acute insulin signaling in the ARC and liver. (**A**) Western blot and densitometric analysis of Hsp10 expression of arcuate nucleus samples of control and Hsp10 KD mice, *n* = 6–7. (**B**) Western blot and densitometric analysis of pAKT (T308) and AKT of arcuate nucleus samples of control and Hsp10 KD mice after insulin injection into vena cava, *n* = 6–7. (**C**) Western blot and densitometric analysis of pAKT (T308) and AKT of liver samples of control and Hsp10 KD mice after insulin injection into vena cava, *n* = 6. *, *p* < 0.05, after two-tailed Student’s *t*-test. All data are presented as mean ± SEM.

To decipher whether reduced Hsp10 expression in the hypothalamus can be an inducer of insulin resistance in vivo, we acutely decreased Hsp10 expression in the arcuate nucleus (ARC) of the hypothalamus. For this, we injected male C57BL/6N mice lentivirus containing shRNA against Hsp10 or a nontarget control into the ARC. This approach resulted in a 27% reduction of Hsp10 in the ARC (Figure 6A). To test insulin signaling, we injected 5 U insulin directly into the vena cava of control and Hsp10 KD mice one week after injection of the lentivirus. This analysis revealed that Hsp10 KD causes hypothalamic insulin resistance evidenced by a 35% reduction in insulin-induced T308 phosphorylation of AKT (Figure 6B). We further investigated the occurrence of astrocyte activation in ARC samples of both groups assessing GFAP expression. This analysis revealed unaltered GFAP protein expression between both groups indicating that the observed insulin resistance is not paralleled by astrocyte reactivity (Figure S4A). Importantly, these mice also exhibited decreased phosphorylation of AKT in the liver (Figure 6C), showing that Hsp10 KD in the ARC also induces hepatic insulin resistance. Mitochondrial dysfunction can induce insulin resistance. To test whether mitochondria deteriorated in the liver of Hsp10 KD mice, we analyzed expression patterns of subunits of the electron transport chain as an indicator of alterations in mitochondrial function and protein abundance. This analysis revealed no differences between tested groups, suggesting that there is no major difference in mitochondrial mass (Figure S4B). Yet, Hsp60 was reduced by 15% in liver samples of Hsp10 KD mice indicating slight alterations in mitochondrial homeostasis (Figure S4C). Since potential folding substrates of Hsp60 were unaltered (Figure S4B), the pathophysiological consequence of this slight reduction of Hsp60 expression on liver function seems to be minor and can presumably not be responsible for altered insulin signaling in the liver of Hsp10 KD mice.

**Figure 7:**
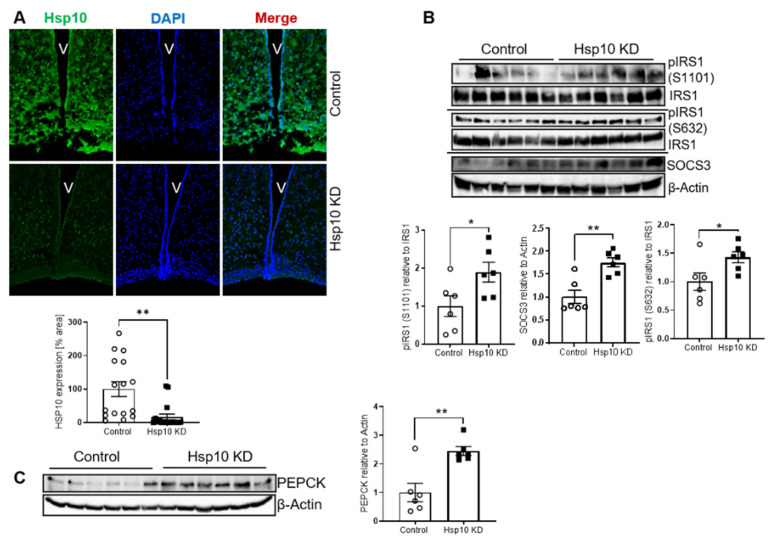
Acute Hsp10 KD in the ARC disturbs insulin action in the ARC and in the liver. (**A**) Representative confocal pictures of immunofluorescent staining of Hsp10 and DAPI of cryo-slices of the brain and analysis of Hsp10 expression, *n* = 16 control and 17 Hsp10 KD mice, 2–3 pictures of each analyzed. (**B**,**C**) Representative Western blot and densitometric analysis of (**B**) pIRS1 (S1101), pIRS1 (S632), SOCS3, and (**C**) of PEPCK of liver samples of control and Hsp10 KD mice. *n* = 6 control *n* = 6 Hsp10 KD. *, *p* < 0.05, **, *p* < 0.01, after two-tailed Student’s *t*-test. All data are presented as mean ± SEM.

To confirm the occurrence of hepatic insulin resistance, we determined markers of insulin resistance in random fed control and Hsp10KD mice in an additional cohort with a verified KD efficiency of about 80% (verification via IHC, Figure 7A). This analysis confirmed that the liver of Hsp10 KD mice exhibited a molecular signature of insulin resistance with 40% increased S1101 and S632 phosphorylation of IRS1 and increased SOCS3 levels (Figure 7B). In addition, liver samples of Hsp10 KD mice exhibited increased PEPCK levels, compared to control, as another indicator of disturbed hepatic insulin action (Figure 7C). In summary, in our acute experimental settings, we describe Hsp10 as a novel regulator of hypothalamic fatty acid metabolism and insulin action in the hypothalamus as well as in the liver.

## 4. Discussion

Mitochondrial function is an integral part of cellular metabolism and health. Deficiencies of mitochondrial proteins in the brain often cause severe mitochondrial dysfunction and are linked to neurological and metabolic diseases. Conversely, mitochondrial proteostasis is a key part of MSR, and the mitochondrial chaperone complex Hsp60/Hsp10 is a crucial part of this response by folding mitochondrial matrix proteins. We show that Hsp10 is reduced in the brains of diabetic mice and that a reduction of chaperone Hsp10 is sufficient to induce mitochondrial dysfunction and cause insulin resistance, a phenotype that was also described for the reduction of Hsp60 in the hypothalamus [11]. These data suggest an association between Hsp10 and insulin signaling in T2D. Additionally, our results clearly show the potency of Hsp10 expression in modulating hypothalamic insulin action since a mild reduction was already sufficient to cause insulin resistance.

Our data show that Hsp10 is mandatory to support metabolic homeostasis in hypothalamic neurons. Hsp10 is an insulin-dependent gene, decreased expressed in hypothalami of different diabetic animal models (this study and [19]) and important for neuronal and brain function [17]. The difference between qPCR and WB data with respect to the variability of Hsp10 expression in db/db mice is so far unknown. Yet, it has been shown that Hsp10 can be sequestered out of mitochondria in certain pathophysiological conditions, which might account for this phenotype [17]. In addition, a basal variation in Hsp10 protein expression has been already described in mice and humans [47,48].

The reduction of Hsp10 activates stress kinases and induces insulin resistance, in addition to altering fatty acid and lipid metabolism with mitochondrial dysfunction, phenotypes also present in neurodegenerative diseases. Indeed, it has been shown that Hsp10 expression is reduced in the putamen of Parkinson’s disease (PD) patients [17], and a missense mutation in HSPE1 is associated with a neurodegenerative disease [18], highlighting the potential protective role of Hsp10 for neuronal health. Since deteriorated insulin action and mitochondrial dysfunction in the brain are involved in this disease development [19,48,49], lack of Hsp10 could be implicated in connecting metabolic disorders and neurodegenerative diseases.

Reduced expression of Hsp10 had a broad effect on fatty acid metabolism with elevated saturated fatty acids and a decrease in monounsaturated fatty acids. Especially, palmitate and stearate were elevated in Hsp10 KD cells, compared to control. It has been shown that high palmitate levels are present in the CSF of obese patients and patients with metabolic syndrome [49,50], and palmitate is able to induce a neuroinflammatory response with hypothalamic insulin and leptin resistance [22,23,51]. Therefore, the Hsp10 KD-induced change in lipid composition might be causative for insulin or leptin resistance. Accordingly, Hsp10 KD neurons display elevated JNK phosphorylation and insulin resistance, phenotypes being induced by palmitate treatment. Furthermore, it has been shown that ICV injection of palmitate blunts insulin and leptin action in the brain and impairs leptin and insulin-induced effects on liver metabolism such as PEPCK regulation [51,52]. This confirms the detrimental effects of elevated hypothalamic palmitate concentrations on insulin and leptin resistance and also reveals phenotypes present in our investigated in vitro and in vivo models of decreased Hsp10 expression. The importance of proper control of hypothalamic fatty acid metabolism is further highlighted by the finding that leptin regulates food intake by altering hypothalamic fatty acid metabolism at the level of acetyl–CoA carboxylase and the generation of malonyl–CoA [53]. Clearly, more research is needed to decipher the effect of Hsp10 KD-induced alterations in fatty acid metabolism on insulin and leptin action.

Neuronal knockdown of Hsp10 also caused alterations in sphingolipid metabolism with an increase in sphingosine-1-phosphate (S1P). Currently, there are controversial data about the beneficial or detrimental effects of S1P on cellular metabolism. On the one hand, it has been shown that palmitate can be metabolized to S1P in hepatocytes [54], which induces insulin resistance [54] and might also induce astrocyte activation [55]. On the other hand, ICV injection of S1P into mice reduces food intake via JAK2/STAT3 dependent signaling [56], signaling molecules being also involved in leptin action and may act neuroprotective in brain injury [57]. Thus far, it remains unknown whether an altered level of S1P is an important mediator of Hsp10 KD-induced cellular effects. Further research should elaborate on the cross talk of Hsp10 with sphingolipid metabolism in regulating cellular metabolism.

Interestingly, the reduction of Hsp10 does not activate the MSR, as opposed to a complementary induction of this response by the lack of mitochondrial proteases [38]. Based on this finding, it seems that altered protein degradation, rather than the inability to properly fold mitochondrial matrix proteins, is the driver of MSR activation. Since Hsp10 reduction causes mitochondrial dysfunction but does not induce the MSR, its loss cannot be compensated. This suggests that Hsp10 expression is crucial to mediate mitochondrial–nuclear communication and reinstate cellular health at least in hypothalamic neurons. Hsp10 reduction causes only mild oxidative stress and decreased mitochondrial activity with a deteriorated cellular metabolism. Slightly impaired mitochondrial function induced by the absence of the mitochondrial protein AIF in hypothalamic POMC neurons is beneficial for metabolism and induces fatty acid oxidation [58]. Our data show that reduced Hsp10 expression in the hypothalamus causes dysregulated fatty acid metabolism and insulin resistance, suggesting an important role of fatty acid homeostasis in the brain to control metabolism. In addition, these data show that cellular consequences of mitochondrial dysfunction cannot be generalized since different dysfunctions can lead to contrasting effects on metabolism.

It has been assumed that the co-chaperone Hsp10 interacts and functions always in combination with its chaperone partner Hsp60. Yet, data are accumulating that both proteins might elicit unique signaling responses. While Hsp60 reduction caused oxidative and inflammation-driven insulin resistance, treating Hsp10 KD neurons with JNK inhibitor or antioxidants did not rescue insulin resistance, suggesting mechanistic differences between Hsp60 and Hsp10 action (this study and [11]). We also observed differences in mitochondrial function in regard to protein expression of subunits of the electron transport chain between Hsp10 and Hsp60 (this study and [11]). This might be due to differences in knockdown efficiencies between Hsp60 and Hsp10, due to the use of different cell lines, or may be based on the finding that Hsp60 can also fold mitochondrial proteins in the absence of Hsp10 [59].

The finding that Hsp10 reduction in the hypothalamus also causes acute liver insulin resistance suggests that hypothalamic Hsp10 function is an interactor of the liver–brain axis. The question remains why a reduction of Hsp10 in the hypothalamus causes acute liver insulin resistance. Hypothalamic inflammation has been shown to cause diet-induced insulin resistance in the liver [60]. This inflammation is present as early as 3 days of HFD feeding and coincides with the reduction of Hsp10 and other mitochondrial genes [19]. We could detect increased activation of the pro-inflammatory kinase JNK and increased mitochondrial–ER contacts in the near absence of Hsp10 in neurons, suggesting a functional relationship between reduced Hsp10 expression and an altered inflammatory response (Figure 3E,F, Figure 5A).

It has been shown that brain insulin action can impact liver metabolism and hepatic insulin action via IL6 and STAT3 signaling [2]. The effect of brain insulin signaling on liver function seems to be also transmitted via the vagus nerve [61]. Accordingly, brain insulin’s effect on liver metabolism is blunted in vagotomized mice [62], showing the importance of functional innervation for brain insulin signaling to alter peripheral metabolism. In *C. elegans*, it has been shown that alteration in mitochondrial protein homeostasis in neurons is able to affect peripheral metabolism via a neuroendocrine signal [63] and even affects mitochondria in the periphery [64], a similar phenotype that we have also observed (Figure S4C). Thus, an altered nerval or neuroendocrine brain–liver communication in Hsp10 KD mice might be responsible for the molecular signature of hepatic insulin resistance. Nevertheless, so far it remains elusive why a decreased expression of Hsp10 in the hypothalamus impacts hepatic insulin sensitivity. Clearly, more research is needed.

Overall, we identified Hsp10 as a crucial regulator of mitochondrial function, fatty acid metabolism, and cellular metabolism in the hypothalamus. Its dysregulation caused insulin hypothalamic resistance and deteriorated fatty acid metabolism with acute hepatic insulin resistance, describing hypothalamic Hsp10 as a potential mediator of the brain–liver axis.

## Data Availability

Data is contained within the article or supplementary material.

## Supplementary Materials

The following are available online at https://www.mdpi.com/article/10.3390/antiox10050711/s1, Suppl Figure S1: Body weight and blood glucose levels of db/db mice. Suppl Figure S2: Hsp10 KD in hypothalamic cells does not impact mitochondrial stress responses or cellular oxidative stress but increases Pgc1α gene expression. Suppl Figure S3: Hsp10 KD in hypothalamic cells does not induce ER stress or apoptosis but impacts fatty acid metabolism and JNK inhibition does not rescue insulin resistance in Hsp10 KD cells. Suppl Figure S4: Hsp10 KD mice do not exhibit increased GFAP expression in the hypothalamus and display only minor effects on markers of mitochondrial function in their livers. Table S1: Primers used for qPCR. Table S2: Mass transitions for HPLC-MS/MS quantification of canonical and deuterated sphingolipids.
