# Supplementary material for: Central Acting Hsp10 Regulates Mitochondrial Function, Fatty Acid Metabolism, and Insulin Sensitivity in the Hypothalamus
Source: Antioxidants (Basel). 2021 Apr 30;10(5):711. doi: 10.3390/antiox10050711 (PMC8145035; doi:10.3390/antiox10050711)
Supplement: Supplementary file 1 [file antioxidants-10-00711-s001.zip › antioxidants-1175876-supplementary.pdf]

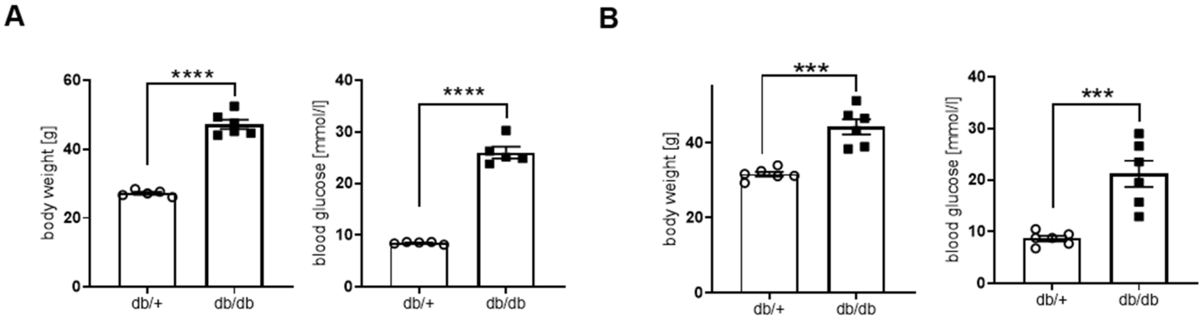

Supplementary Figure S1. Body weight and blood glucose levels of db/db mice. **(A)** Body weight and blood glucose levels of 14-week-old male db/+ and db/db mice used for Western blot analysis (Figure 1C, D). **(B)** Body weight and blood glucose levels of 14-week-old male db/+ and db/db mice used for Western blot analysis of Hsp10 and mRNA analysis of Hsp10 (Figure 1A,B). ***, $p < 0.001$, ****, $p < 0.0001$ after two-tailed Student's t-test. All data are presented as mean ± SEM.

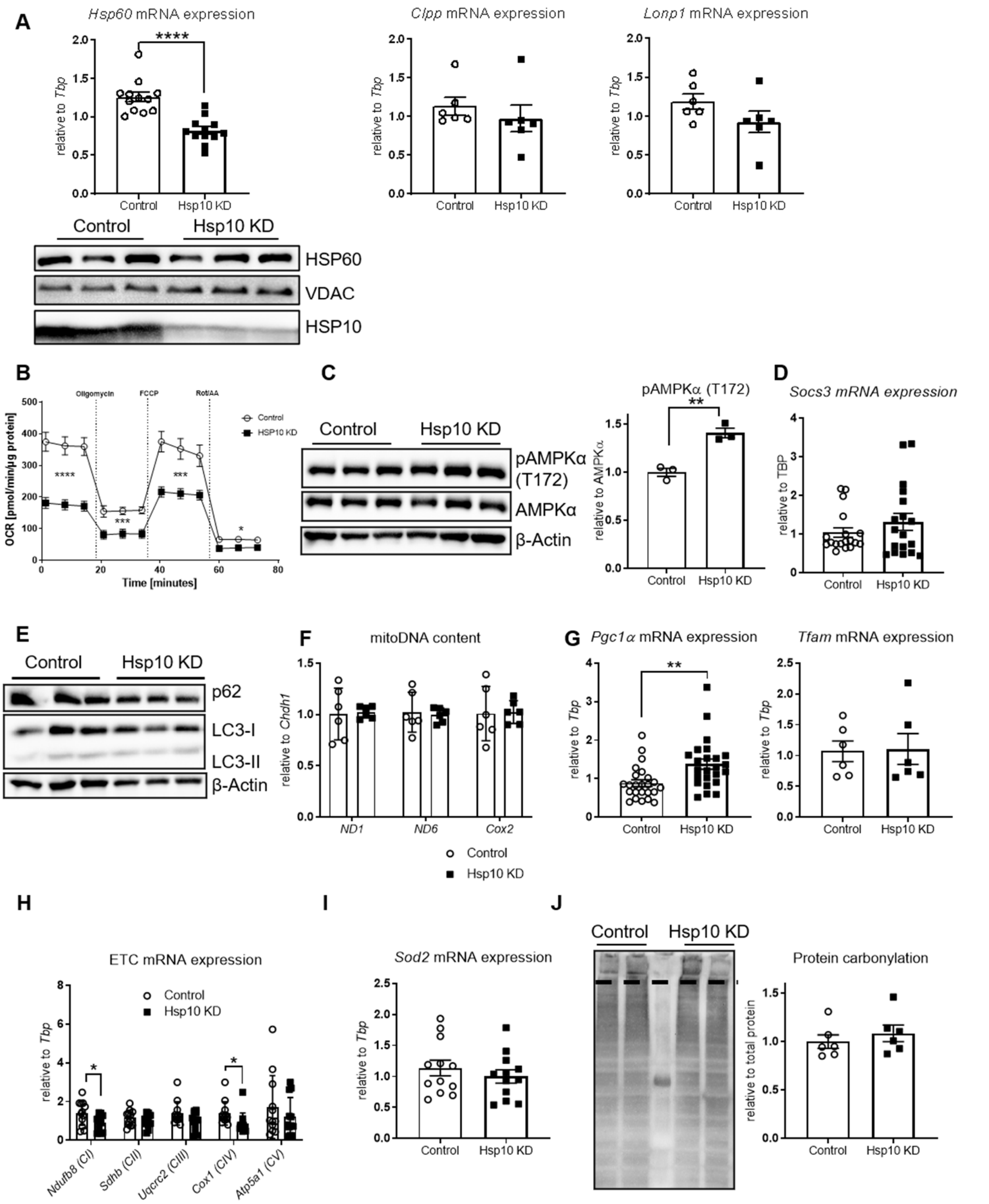

Supplementary Figure S2. Hsp10 KD in hypothalamic cells does not impact mitochondrial stress responses or cellular oxidative stress, but increases Pgc1α gene expression. **(A)** (top left) mRNA expression and (bottom left) protein expression of Hsp60 of control and Hsp10 KD cells with additional verification of Hsp10 KD on protein level. VDAC as control for mitochondrial content. Representative blot of three independent experiments, n = 3. RT-PCR n = 18. (right) mRNA expression of Clpp and Lonp1 of control and Hsp10 KD cells, n = 6. **(B)** Original bioenergetic profile of the seahorse data shown in Figure 2D. **(C)** Protein expression and densitometric analysis of pAMPK (T172) of control and Hsp10 KD. β-Actin served as a loading control n = 3. Representative blot from

three independent experiments. **(D)** mRNA expression of Socs3 of control and Hsp10 KD cells (n = 18). **(E)** Protein expression of p62, LC3 I, and II for control and Hsp10 KD cells (n = 3). **(F)** Mitochondrial DNA (mitoDNA) content of control and Hsp10 KD cells, n = 12. **(G)** mRNA expression of mitochondrial biogenesis marker Pgc1α (n = 24) and mitochondrial genome transcription activator Tfam (n = 6) of control and Hsp10 KD cells. **(H)** mRNA expression of ETC complex subunits CI-CV of control and Hsp10 KD cells, n =18. **(I)** mRNA expression of Sod2 of control and Hsp10 KD cells, n = 12. **(J)** Western blot and densitometric analysis of total protein carbonylation of control and Hsp10 KD cells. Representative blot from three different experiments. *, $p < 0.05$, ***, $p < 0.001$, **, $p < 0.01$, ****, $p < 0.0001$ after two-tailed Student's t-test. All data are presented as mean ± SEM.

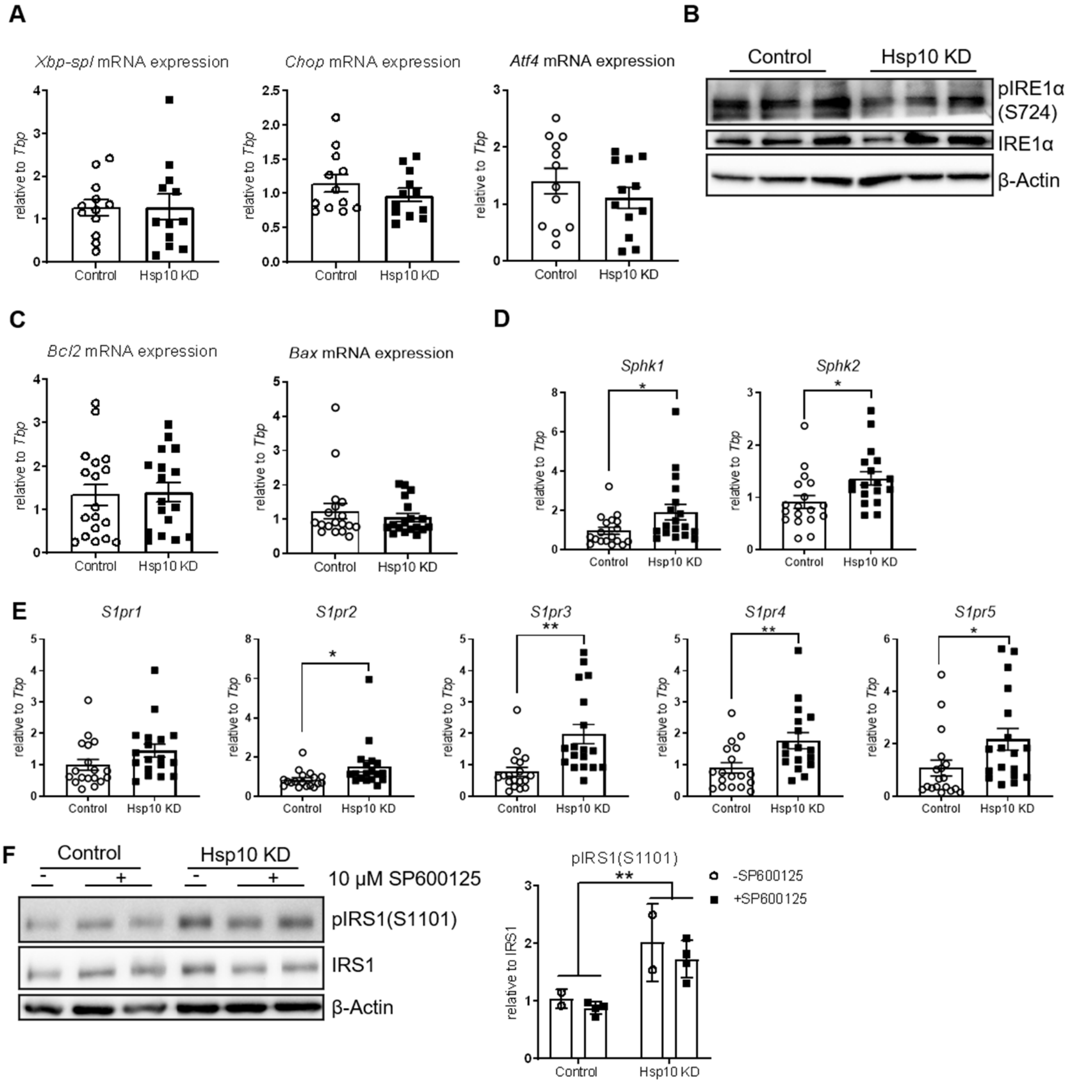

Supplementary Figure S3. Hsp10 KD in hypothalamic cells does not induce ER stress or apoptosis but impacts fatty acid metabolism and JNK inhibition does not rescue insulin resistance in Hsp10 KD cells. **(A)** mRNA expression of ER stress markers namely Xbp-spliced, Chop and Atf4 of control and Hsp10 KD cells (n = 12) and **(B)** protein expression of pIRE1α (S724), n = 3. Representative Western blot of two independent experiments. **(C)** mRNA expression of apoptotic markers Bcl2 and Bax of control and Hsp10 KD cells, n = 17–18. **(D)** mRNA expression of sphingosine kinase 1 and 2 (Sphk1, Sphk2) of control and Hsp10 KD cells, n = 17–18. **(E)** mRNA expression of Sphingosine-1-phosphate receptor isoforms 1–5 (S1pr1–5) of control and Hsp10 KD cells, n = 17–18. **(F)** Representative Western blot and pooled densitometric analysis of pIRS1(S1101) in control and Hsp10 KD cells after 19 h treatment of JNK inhibition with 10µM SP600125, n = 4. *, $p < 0.05$, **, $p < 0.01$ after two-tailed Student's t-test. All data are presented as mean ± SEM.

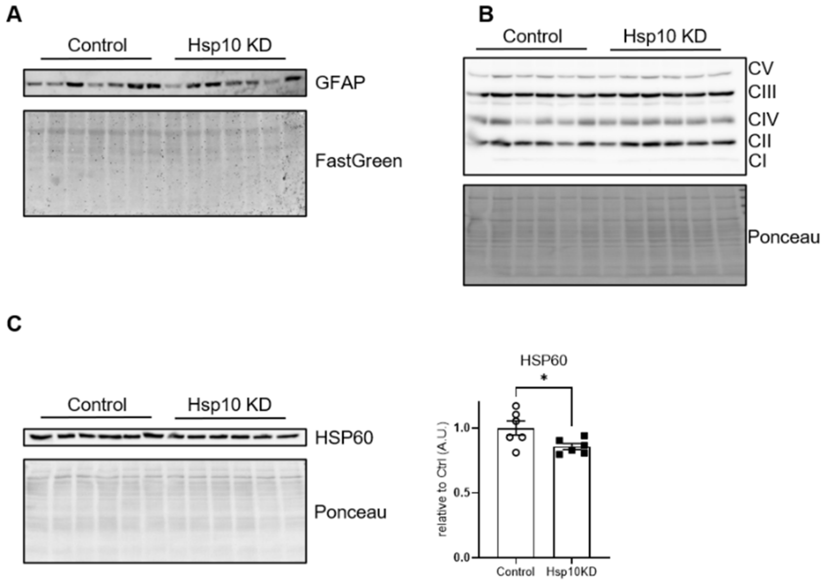

Supplementary Figure S4. Hsp10 KD mice do not exhibit increased GFAP expression in the hypothalamus and display only minor effects on markers of mitochondrial function in their livers. **(A)** Western blot of GFAP expression of arcuate nucleus samples of control and Hsp10 mice, FastGreen stain served as loading control, $n = 7$. **(B)** Western blot of mitochondrial OXPHOS complex subunits of CII-CV of liver samples of control and Hsp10 mice, Ponceau stain served as loading control, $n = 6$. **(C)** Western blot and densitometric analysis of Hsp60 of liver samples of control and Hsp10 mice, Ponceau stain served as loading control, $n = 6$. *, $p < 0.05$, after two-tailed Student's t-test. All data are presented as mean ± SEM.

Supplementary Table S1

Primer	Forward	Reverse
Acadl	GGTGGAAAACGGAATGAAAGG	GGCAATCGGACATCTTCAAAG
Acadm	TGTTAATCGGTGAAGGAGCAG	CTATCCAGGGCATACTTCGTG
Acads	CCTGGGATGGGCTTCAAAATAG	GGTTCTCGGCATACTTCACAG
Atf4	CCTGAACAGCGAAGTGTTGG	TGGAGAACCCATGAGGTTTCAA
Atp5a1 (CV)	CATTGGTGATGGTATTGCGC	TCCCAAACACGACAACTCC
Bax	GGAGACACCTGAGCTGACCT	CAGCAATCATCCTCTGCAGCTC
Bcl2	GTGTGGAGAGCGTCAACAGG	CACAAAGGCATCCCAGCCTC
Chdh1	GCACAGTGGCCCTTAAATGT	CCCTGCCTAAAATACCGTGA
Clpp	ATATACTCGAGGCTGTTGCG	CCACCTGGGCTGTTGATATAC
Cox1 (CIV)	GACACACGAGCTTACTTTAC	GCTATGATAGCAAACACTGC
Cox2	CCTGGTGAACTACGACTGCT	GAATAACCCTGGTCGGTTTG
Ddit3 (CHOP)	CTGCCTTTCACCTTGGAGAC	CGTTTCCTGGGGATGAGATA
h_HSP10	GTATTGGTTGAAAGGAGTGCTG	CACGCTAACTGGTTGAATCTCT
h_TBP	GCCATAAGGCATCATTGGAC	AACAACAGCCTGCCACCTTA
Hprt	GCAGTCCCAGCGTCGTG	GGCCTCCCATCTCCTTCAT
Hspd1 (Hsp60)	AGTGTTCAGTCCATTGTCCC	TGACTGCCACAACCTGAAG
Hspe1 (Hsp10)	CTGCCGAAACTGTAACCAAAG	TCTCCAACTTTCACACTGACAG
Lonp1	CTTCCGTTTCAGTGTTGGTG	GGGTTCTCTGTCTTGGTCTTC
Mfn1	CATTGCGTTTCGGTTTTCCC	GAAGGAGCAGTAGGAGTTGAAG
Mfn2	ATCAGTTACACCGGCTCTAAC	GCCTCGACTTTCTTGTTCATG
Nd1	GGATCCGAGCATCTTATCCA	GGTGGTACTCCCGCTGTAAA
Nd6	ATTAAACAACCAACAAACCCAC	TTTGGTTGGTTGTCTTGGGTT
Ndufb8 (CI)	ACATCTCTTCGGCTTTGTGG	CAGGCTCTTTGGTAGGATCAC
Opa1	GTGTGCTGGAAATGATTGCTC	TGGTGAGATCAAATTCCCGAG
Pgc1α	AGCCGTGACCACTGACAACGAG	GCTGCATGGTTCTGAGTGCTAAG
pLKO.1	CATATAGTATGGGCAAGCAGGG	CTGTCTGAAGGGATGGTTGTAG
S1pr1	TTCTCATCTGCTGCTTCATCATCC	GGTCCGAGAGGGCTAGGTTG
S1pr2	TTACTGGCTATCGTGGCTCTG	ATGGTGACCGTCTTGAGCAG
S1pr3	GCGTGTTCCTTCTGATTGG	GCAAGATGGTAGAGCAGTC
S1pr4	CTGTCAGGGACTCGTACC	CGTGAAGAGCAGACTGAAG
S1pr5	CCAACAGCTTGCAGCGATCCCC	GGTTGCTACTCCAGGACTGCCG
Sdhb (CII)	ACCCCTTCTCTGTCTACCG	AATGCTCGCTTCTCCTTGTAG
Sod2	TGCTCTAATCAGGACCCATTG	CATTCTCCCAGTTGATTACATTCC
Sphk1	AAAATACTGAGAAACTCGGTCGG	GCATCGCTTCTTAAAGTCCAGA
Sphk2	CACGGCGAGTTTGGTTCCTA	CTTCTGGCTTTGGGCGTAGT
Tbp	CTGGAATTGTACCGCAGCTT	ATGATGACTGCAGCAAATCG
Tfam	CACCCAGATGCAAAACTTTCAG	CTGCTCTTTATACTTGCTCACAG
Uqcrc2 (CIII)	TTCCAGTGCAGATGTCCAAG	CTGTTGAAGGACGGTAGAAGG
Xbp-spliced	TGCTGAGTCCGCAGCAGGTG	GCTGGCAGGCTCTGGGGAAG

Supplementary Table S2. Mass transitions for HPLC-MS/MS quantification of canonical and deuterated sphingolipids.

Compound	Precursor ion (*m*/*z*)		Product ion (*m*/*z*)		Collision energy (eV)
	canonical	deuterated	canonical	deuterated	
**Sph**	300.3	303.3	282.3	285.3	8
dhSph	302.3	305.3	284.3	287.3	12
S1P	380.3	383.3	264.3	267.3	16
C16:0 Cer	520.5	523.5 / 526.5	264.3	267.3	25
C18:0 Cer	548.5	551.5	264.3	267.3	25
C20:0 Cer	576.6	579.6	264.3	267.3	25
C22:0 Cer	604.6	607.6	264.3	267.3	25
C24:0 Cer	632.6	635.6	264.3	267.3	25
C24:1 Cer	630.6	633.6	264.3	267.3	25
C16:0 dhCer	540.5	543.6 / 546.6	522.5	525.5 / 528.5	15
C18:0 dhCer	568.5	571.5	550.5	553.5	15
C20:0 dhCer	596.6	599.6	578.6	581.5	15
C22:0 dhCer	624.6	627.6	606.6	609.6	15
C24:0 dhCer	652.7	655.7	634.6	637.6	15
C24:1 dhCer	650.6	653.6	632.6	635.6	15
C16:0 SM	703.6	706.6 / 709.6	184.1	184.1	25
C18:0 SM	731.6	734.6	184.1	184.1	25
C20:0 SM	759.6	762.6	184.1	184.1	25
C22:0 SM	787.7	790.7	184.1	184.1	25
C24:0 SM	815.7	818.7	184.1	184.1	25
C24:1 SM	813.7	816.7	184.1	184.1	25
C16:0 dhSM	705.6	708.6 / 711.6	184.1	184.1	25
C18:0 dhSM	733.6	736.6	184.1	184.1	25
C20:0 dhSM	761.6	764.6	184.1	184.1	25
C22:0 dhSM	789.7	792.7	184.1	184.1	25
C24:0 dhSM	817.7	820.7	184.1	184.1	25
C24:1 dhSM	815.7	818.7	184.1	184.1	25

The following mass transitions were used for internal standard compounds: *m*/*z* 307.3 → 289.3 for d_7-Sph (8 eV), *m*/*z* 309.3 → 291.3 for d_7-dhSph (12 eV), *m*/*z* 387.3 → 271.3 for d_7-S1P (16 eV), *m*/*z* 534.5 → 264.3 for C17:0 Cer (applied for all Cer and dhCer species) and *m*/*z* 734.8 → 184.1 for C16:0 d_{31}-SM (applied for all SM and dhSM species).

For C16:0 (dh)Cer and (dh)SM species d_3 and d_6-labeled analogues might be formed after cell stimulation with palmitate-d_3. All other sphingolipid species form d_3-labeled analogues only. .

Sph, sphingosine; dhSph, dihydrosphingosine; S1P, sphingosine 1-phosphate; Cer, ceramide; dhCer, dihydroceramide; SM, sphingomyelin; dhSM, dihydrosphingomyelin.

Supplementary Methods

Cell Culture Stimulation Experiments

CLU-183 cells were stimulated with 10µM SP600125 (Sigma-Aldrich, St. Louis, MO, USA) for 19h in full media.
